# Effectiveness of Multidomain Dormitory Environment and Roommate Intervention for Improving Sleep Quality of Medical College Students: A Cluster Randomised Controlled Trial in China

**DOI:** 10.3390/ijerph192215337

**Published:** 2022-11-20

**Authors:** Man Li, Qing Han, Ziqi Pan, Kailu Wang, Junqing Xie, Bang Zheng, Jun Lv

**Affiliations:** 1 Sun Yat-sen University Cancer Center, No. 651 Dongfeng East Road, Yuexiu District, Guangzhou 510060, China; 2 Department of Epidemiology & Biostatistics, School of Public Health, Peking University, Beijing 100191, China; 3 Department of Social Policy and Intervention, University of Oxford, Oxford OX1 2ER, UK; 4 Centre for Health Systems and Policy Research, JC School of Public Health and Primary Care, The Chinese University of Hong Kong, Hong Kong 999077, China; 5 Centre for Statistics in Medicine and National Institute for Health and Care Research Biomedical Research Centre Oxford, NDORMS, University of Oxford, Oxford OX3 7LD, UK; 6 Department of Non-Communicable Disease Epidemiology, London School of Hygiene & Tropical Medicine, London WC1E 7HT, UK; 7 Peking University Center for Public Health and Epidemic Preparedness & Response, Peking University, No. 38 Xueyuan Road, Haidian District, Beijing 100191, China

**Keywords:** sleep quality, medical students, dormitory environment, roommate, intervention

## Abstract

Medical students are vulnerable to sleep disorders, which could be further exaggerated by poor dormitory environment and roommate behaviour. However, there is little evidence of whether dormitory environment intervention is effective in improving the sleep quality of medical college students in developing countries. The present study aimed to evaluate the effects of a comprehensive multidomain intervention on dormitory environment and roommate behaviour among medical college students in China. In this cluster randomised controlled trial, a total of 106 dormitories (364 students) were randomly allocated into an intervention group (55 dormitories, 193 students) and a control group (51 dormitories, 171 students). The intervention group received a three-month intervention with multiple components to improve or adapt to sleep environments in dormitories; the control group received no intervention. Primary and secondary outcomes were measured at study enrolment and three months later for both groups. The linear mixed-effects models showed that, compared with the control group, the intervention was associated with a significantly decreased Pittsburgh Sleep Quality Index (β = −0.67, *p* = 0.012), and a marginally significant effect on reducing roommates’ influence on sleep schedule (β = −0.21, *p* = 0.066). Students in the intervention group rated “making dormitory sleep rules” and “wearing eye masks” as the most effective intervention measures. These findings could contribute to the limited body of scientific evidence about sleep intervention in Chinese medical students and highlight the importance of dormitory sleep environments in maintaining sleep quality.

## 1. Introduction

Sleep quality has a great impact on people’s physical and mental state, which could affect a wide array of social behaviours and health outcomes. Good sleep quality has been proven to be associated with better academic performance among university students [1]. However, many previous studies reported that the sleep quality of medical students was generally poor across different countries [2,3], and the possible causes may include a large academic load [4], a high level of clinical work intensity and mental pressure [5] and improper sleep hygiene practices of some students [6]. A systematic review and meta-analysis in 2022 found that the prevalence of sleep problems among Chinese medical students was 27.4% and there were significant differences by regions and educational backgrounds [7], which revealed a realistic public health problem that could not be ignored. Sleep can influence cellular metabolic stress in both brain and peripheral tissues [8]. A systematic review in 2015 [9] of experimental evidence reported that sleep can directly promote and strengthen the liver antioxidant system, which is blocked when sleep deprivation occurs, and sleep deprivation could increase the oxidative stress of the heart. Another review article [10] showed that sleep deprivation may alter protein synthesis and synaptic plasticity through oxido-inflammatory mechanisms.

Given the current situation of prevalent sleep problems among medical students, a systematic review in 2020 advocated for an urgent intervention aiming at improving sleep quality of medical students [11]. Up to now, although there have been many studies on sleep quality of medical students and its related factors, there are relatively few research on sleep intervention, especially in developing countries. A study conducted among medical students in Hubei Province of China reported that after a 3 km brisk walking exercise intervention for more than 20 times within 6 weeks, the sleep quality of the intervention group was improved to a certain extent [3]. Cognitive behavioural therapy could also be an effective way to improve sleep. Zhong et al. [12] reported that cognitive behaviour intervention based on WeChat, a social media platform in China, can significantly improve the sleep quality of medical students. Positive psychological interventions have also been proposed to adjust students’ unhealthy sleep behaviours and improve their sleep quality [13]. In addition, Brubaker et al. [14] found that a brief intervention by making a two-week sunrise alarm clock protocol with electronic device removal was effective in improving sleep quality among medical students.

In addition to the potential intervention approaches mentioned above, dormitory sleep environment may be a promising target for intervention. Since most university/college students in China live in residence halls (with shared rooms) on the campus, their sleep schedule and sleep quality could easily be influenced by roommate behaviour and dormitory environment in the evening. However, little attention has been paid to this research area and there lacks targeted research on sleep behaviour interactions between roommates in dormitories and relevant intervention strategies.

Therefore, the present study aimed to examine the effectiveness of dormitory sleep environment intervention using a cluster randomised controlled trial (RCT) in a medical college in Beijing, China. We designed a comprehensive multidomain intervention approach to help students improve or adapt to their dormitory sleep environments with the purpose of improving their sleep quality.

## 2. Methods

### 2.1. Participants

Participants were undergraduate students living in residence halls at a medical college in Beijing. The inclusion criteria were: long-term residence in the dormitory (defined as 4 days or more per week on average during the term time); 4 or 3 people living in a dormitory (i.e., quadruple-occupancy or triple-occupancy rooms); and all members of the dormitory agreed to participate in the study and signed the informed consent form. Participants who did not complete the baseline survey at recruitment were excluded from this study. This study was conducted between 2014 and 2015. The study protocol had been reviewed and approved by the Student Innovative Experiment Program Committee of Peking University Health Science Center and the Biomedical Ethics Committee of Peking University before implementation (Approval number: IRB00001052-14053).

### 2.2. Cluster Randomisation

We recruited 418 undergraduates of year 2–4 from 110 dormitories (the first-year undergraduates resided on a different campus), of whom 364 students from 106 dormitories fulfilled the inclusion criteria and were included in this study. Given the study design of cluster RCT, we considered dormitory as the cluster and randomised the 106 dormitories into an intervention group and a control group using random numbers generated by the SPSS software. The randomisation was stratified by sex and grade to avoid imbalanced randomisation results. After randomisation, there were 55 dormitories (193 students) in the intervention group and 51 dormitories (171 students) in the control group.

### 2.3. Intervention Procedures

The intervention setting was the college dormitory. The intervention focused on the night-time behaviour habits and interactions between roommates, such as roommates’ sleep schedule, work and rest habits, dormitory light off time, noises generated by roommates and dormitory collective activities, but did not include the objective physical environment such as hardware facilities or temperature in the dormitory.

The intervention lasted three months and included the following components: (1) health education on the importance of sleep, sleep hygiene, and dormitory sleep environment (through face-to-face small-group health education sessions [30 min per session, 1 session per month] and handing out a brochure on improving sleep environment and protecting roommates’ sleep); (2) making dormitory sleep rules (through the health education and a poster suggested to be hung on their doors which includes a column of the school calendar and a column for writing down the sleep rules), such as light off after 11 p.m., no loud conversation after at least one roommate go to bed, wearing earphone when listening to music/playing computer games at bedtime; (3) providing earplugs to reduce noise during sleep; and (4) providing eye masks to reduce light during sleep. The dormitories allocated to the control group received no intervention. We chose the 3-month intervention time because we want to keep the intervention within one school term to reduce the loss of follow-up and the possibility that some students would move to hospitals for training/internship in the longer term.

### 2.4. Outcome Measures

The primary outcome of this study was the total score of the Chinese version of Pittsburgh Sleep Quality Index (PSQI) [15], roommates’ influence on sleep schedule, dormitory environment influence on sleep quality, and self-rated dormitory sleep environment. PSQI is a validated scale which assesses sleep quality and disturbance over a 1-month period and had been translated and adapted into Chinese. The scale consists of 19 self-reported items and could be classified into seven subdimensions: subjective sleep quality, sleep latency, sleep duration, habitual sleep efficiency, sleep disturbances, use of sleep medications, and daytime dysfunction due to sleepiness. All subdimensions yield a score from 0 to 3 and could be summed to yield a total score ranging from 0 to 21, with higher scores indicating worse sleep quality. This scale had been shown to be an effective sleep screening tool with good reliability and validity among Chinese medical college students [16]. The other three items were measured on a 5-point Likert scale: in the recent month, (1) to what extent was your sleep schedule influenced by your roommates? (from 1 “not at all” to 5 “a lot”); (2) to what extent was your sleep quality influenced by dormitory sleep environment? (from 1 “not at all” to 5 “a lot”); (3) how do you rate your dormitory sleep environment, including roommate behaviour, the overall atmosphere, noise and light pollution, etc. (from 1 “very poor” to 5 “very good”).

Secondary outcomes included the seven subdimensions of the PSQI, self-reflection of potential disturbance to roommate’s sleep, reaction to roommate’s reminder of disruptive behaviour when they were trying to sleep, dormitory conflict due to sleep disturbances, roommate relationship, and self-rated importance of sleep. Detailed items are presented in Supplementary Materials. All the primary and secondary outcomes were measured at enrolment and three months later for both groups.

In addition, a short post-intervention survey was administered to participants in the intervention group to collect their feedback and subjective evaluation of the intervention program (Supplementary Materials).

### 2.5. Statistical Analyses

Data analysis was conducted by a statistician (QH) blinded to the group allocation status. Baseline characteristics of participants and outcome variables were described by group. The linear mix-effects model (random-intercept) was used to evaluate the intervention effect on outcome measure, with room number and participant ID as two nested random effects in consideration of the clustered design and the repeated measurements. In each regression model, the dependent variable was a specific outcome variable; the independent variables were group (intervention vs. control), time points (pre-test vs. post-test), and the interaction term of group and time which reflects the intervention effect. All models were adjusted for age, grade, sex, and academic stress as covariates. Within-group analyses by paired *t* tests were then conducted to characterise the change in outcome variables (i.e., pre–post difference) in intervention group and control group. A descriptive analysis of the feedback from the intervention group regarding the intervention procedures was also conducted.

Several sensitivity analyses were conducted to assess the robustness of the main findings: (1) using linear mixed-effects models with no covariates; (2) additionally adjusting for major (seven categories), experience of living in the dormitory in high school, one-child family, monthly living expenses (reflecting economic status), self-rated physical health, baseline PSQI total score, and baseline level of self-rated importance of sleep.

All statistical analyses were performed using R (version 4.3.1, R Core Team) and all statistical tests were two-sided; *p* < 0.05 was used as the significance threshold.

## 3. Results

Table 1 presents the main baseline characteristics of 193 participants in the intervention group and 171 participants in the control group. No substantial differences in these variables were detected between the two groups.

**Figure 1:**
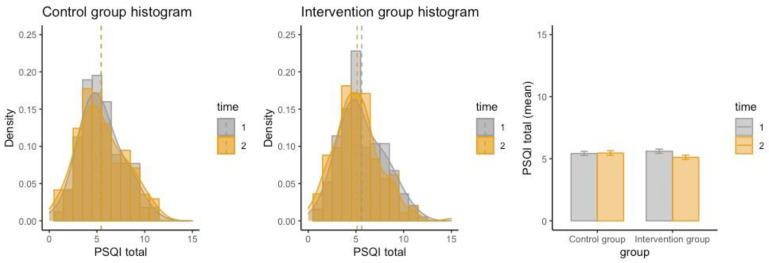
Distributions of pre- and post-intervention PSQI total scores in the two groups. Note. PSQI = Pittsburgh Sleep Quality Index. Time 1 refers to enrolment date; time 2 refers to study end date. The error bars in the bar plot refer to standard errors.

The pre- and post-intervention levels of the four primary outcome variables and 12 secondary outcome variables are shown in Table 2. Results of the linear mixed-effects model showed that the change in PSQI total score in the intervention group was significantly larger on average than that in the control group (β = −0.67, *p* = 0.012; Table 3). As visualised in Figure 1, the mean PSQI total score decreased in the intervention group after intervention (*p* = 0.011; lower PSQI means better sleep), but no change in the PSQI total score was observed in the control group (*p* = 0.643). The intervention effect on reducing roommates’ influence on sleep schedule was of borderline statistical significance (β = −0.21, *p* = 0.066; Table 3); further within-group analysis showed that roommates’ influence on sleep schedule was significantly decreased in the intervention group (*p* = 0.028) but not in the control group (*p* = 0.761). Results of the linear mixed-effects model showed no significant intervention effects on self-rated dormitory sleep environment or dormitory environment influence on sleep quality (Table 3), but the within-group analysis detected a significant decrease in dormitory environment influence on sleep quality in the intervention group (0.044) but not in the control group (*p* = 0.278).

Table 4 shows the intervention effect on secondary outcome variables. The intervention was significantly associated with a decrease in the PSQI component of subjective sleep quality (β = −0.22, *p* = 0.008), better reaction to roommate’s reminder of disruptive behaviour (β = −0.22, *p* = 0.038) and improved roommate relationship (β = 0.20, *p* = 0.006). There was no evidence of significant intervention effects on other secondary outcomes (Table 4). Sensitivity analyses of the intervention effects showed consistent results with the main analysis (Supplementary Materials). No harms or unintended effects were observed during the study.

After the intervention, 55.4% and 3.6% of participants in the intervention group reported that their dormitory sleep environment was improved “to some extent” or “substantially”, respectively, while 39.9% and 1.5% reported “no change” or “worse environment”. Of the participants in the intervention group, 41.7% and 4.7% reported that their sleep quality was improved “to some extent” or “substantially”, respectively, while 50.0% and 3.6% reported “no change” or “worse to some extent”. Of the participants 62.0% and 10.9% reported that the intervention was “acceptable” or “highly acceptable” to them, while 20.8% and 6.3% reported “reluctantly acceptable” or “not acceptable”, respectively. Among the four intervention components, 44.8% of participants reported that making dormitory sleep rules was an effective measure, 43.2% reported wearing eye mask was effective, and 36.5% and 20.3% thought wearing earplugs or sleep education program was effective. Specifically, we found that 25.1% and 22.2% of participants in the control group reported that their dormitory had specific sleep rules before and after the study, respectively, while this proportion increased from 21.8% to 40.6% in the intervention group.

## 4. Discussion

In this cluster randomised controlled trial, we assessed the effect of a dormitory-based multidomain intervention (targeting dormitory environment and roommate influence) on sleep quality among 364 medical college students in China. There was a significant association between the intervention and a decreased Pittsburgh Sleep Quality Index (reflecting improved sleep quality), and suggestive evidence for the intervention effect on reducing roommates’ influence on sleep schedule. We also observed that the most effective intervention measures reported by students in the intervention group were making dormitory sleep rules and wearing eye mask.

The sleep quality of medical students could be affected by a range of physiological, social psychological, and environmental factors. Sexton-Radek et al. [17] reported that noise and light were significant sleep disturbances in the sleep environment of American college students living in residential halls, which was consistent with our previous findings on the associations between dormitory environment and sleep quality in Chinese medical college students [18]. Experimental research also found that night light exposure can reduce the sleepiness of college students and is related to the secretion time of melatonin in the human body [19]. Therefore, this study focused on intervening with roommates’ sleep schedule, work and rest habits, dormitory light off time, noises generated by roommates and dormitory collective activities to reduce the impact of poor dormitory environment on sleep quality. Our results confirmed that the three-month dormitory environment intervention was effective in the study population.

Making dormitory sleep rules is an important and innovative component of the intervention and may improve all roommates’ awareness of maintaining a good sleeping environment in the dormitory, and even restrict the inappropriate behaviour of roommates at bedtime. In addition, wearing eye masks and earplugs can directly reduce the degree of light exposure and noise exposure when falling asleep and during sleep. A cross-over clinical trial [20] tested the effect of earplugs and eye mask on the perceived sleep quality of patients in intensive care unit, and showed that the patients’ nocturnal sleep quality enhanced in the night of wearing earplugs and eye mask. Hu et al. [21] found that earplugs and eye masks can play a positive role in improving sleep quality, promoting hormone balance and improving the level of REM sleep and nocturnal melatonin level in healthy subjects.

There is a growing body of evidence [22] that poor sleep quality measured by the PSQI is associated with stress levels [5,22,23,24]. A high level of stress is a major predictor and contributor to poor sleep quality [5]. A previous study [25] reported that the hypothalamus–pituitary–adrenal axis plays a major role in stress and cardiovascular disease, and sleep might also be closely related to this process. Vgontzas et al. [26] found that fatigue-inducing pro-inflammatory cytokines (interleukin-6 and tumour necrosis factor alpha) are negatively linked to the quantity and quality of sleep. Inflammation can cause unhealthy status and further lead to worse sleep quality. A cross-sectional study [5] found 53.2% of medical students reported psychological stress, and demonstrated a significant association between stress and sleep quality among medical students. In fact, a considerable proportion of medical students choose to reduce their sleep time to extend the time available for study, in order to obtain a good academic performance; consequently, they become sleep-deprived and stressed [27]. In the present study, the comprehensive multidomain intervention may also promote the medical college students to create quiet dormitory environment and more harmonious roommate relationship, and develop more regular living and learning habits, which to some extent make medical students feel psychologically relaxed and less stressed, thus having a higher possibility to obtain a good sleep quality. Future sleep interventions with detailed measurement of mental health and axis 1 disorders are needed to elucidate the mechanisms.

The present study also had some potential limitations. Firstly, the representativeness of the study population is limited, due to only covering one medical college in Beijing. Secondly, due to the practical restrictions and feasibility issues, there is no intervention component on the objective physical environment in the dormitory, and lack of objectively measured sleep quality and recorded night-time behaviour of roommates, though our measurements of dormitory sleep environment covered a wide range of subjective aspects. Thirdly, given that the dormitories under intervention may be close to the dormitories in the control group, there could be treatment contamination which may have led to the underestimation of intervention effects. Further large-scale RCTs on dormitory-based sleep intervention are required to validate our findings. We expect the results from this study will contribute to the design of appropriate comprehensive sleep intervention guidelines to achieve the purpose of improving sleep quality of medical students and students in other subjects.

## 5. Conclusions

In conclusion, we found that comprehensive intervention on dormitory sleep environment and roommate behaviour was effective in improving the sleep quality of medical students. An urgent appeal for schools and students should be made to emphasise sleep problems from the aspect of dormitory environment, and to actively create a good dormitory sleep environment and avoid sleep conflicts among roommates. Our findings could contribute to the limited scientific evidence about the sleep behaviour intervention and the influence of dormitory sleep environment on the sleep quality of college students.

## Figures and Tables

**Table 1:** Description of participant characteristics.

Characteristics	Control Group	Intervention Group
N	171	193
Age, mean (SD)	20.1 (1.3)	20.1 (1.3)
Sex (female %)	59.6%	54.4%
Grade (%)		
Year 2	39.2%	38.9%
Year 3	39.2%	42.0%
Year 4	21.6%	19.2%
One-child family (%)	58.5%	65.1%
High-school experience of living in dormitory (%)	68.4%	65.8%
Academic stress level, mean (SD) *	3.41 (0.95)	3.35 (0.89)
Self-rated physical health, mean (SD) *	2.01 (0.61)	2.01 (0.71)

Note. SD = standard deviation. * these items were measured on a 1–5 scale.

**Table 2:** Pre- and post-intervention level of primary and secondary outcome variables in both groups.

Outcome Variables	Control Group				Intervention Group			
	Mean_pre_	SD_pre_	Mean_post_	SD_post_	Mean_pre_	SD_pre_	Mean_post_	SD_post_
Primary outcomes								
PSQI total score	5.43	2.21	5.46	2.46	5.61	2.35	5.12	2.43
Roommates’ influence on sleep schedule	2.37	0.99	2.39	0.92	2.58	0.98	2.41	0.88
Dormitory environment influence on sleep quality	2.42	0.96	2.33	0.87	2.47	0.94	2.32	0.85
Self-rated dormitory sleep environment	3.64	0.97	3.63	0.86	3.56	0.93	3.66	0.86
Secondary outcomes								
PSQI—Subjective sleep quality	0.96	0.64	1.11	0.71	1.10	0.69	1.05	0.68
PSQI—Sleep latency	0.98	0.83	0.95	0.81	0.99	0.82	0.96	0.87
PSQI—Sleep duration	0.40	0.64	0.36	0.58	0.48	0.61	0.36	0.58
PSQI—Sleep efficiency	0.15	0.39	0.19	0.45	0.18	0.44	0.13	0.44
PSQI—Sleep disturbance	0.83	0.51	0.91	0.54	0.87	0.48	0.87	0.50
PSQI—Use of sleep medication	0.04	0.30	0.12	0.46	0.01	0.07	0.03	0.19
PSQI—Daytime dysfunction	2.06	0.76	1.81	0.86	1.98	0.85	1.72	0.91
Self-reflection of potential disturbance to roommate’s sleep	3.81	0.96	3.74	0.96	4.01	0.79	3.78	0.91
Reaction to roommate’s reminder of disruptive behaviour	1.50	0.81	1.73	0.89	1.66	0.85	1.68	0.84
Dormitory conflict due to sleep disturbances	1.40	0.65	1.26	0.6	1.35	0.58	1.31	0.65
Roommate relationship	4.46	0.66	4.30	0.71	4.19	0.69	4.23	0.70
Sleep importance	4.71	0.65	4.64	0.59	4.68	0.57	4.60	0.65

Note. Mean_pre_ = Pre-intervention mean; Mean_post_ = Post-intervention mean; SD_pre_ = Pre-intervention standard deviation; SD_post_ = Post-intervention standard deviation; PSQI = Pittsburgh Sleep Quality Index.

**Table 3:** Linear mixed-effects models of intervention effect on primary outcomes.

Primary Outcomes		β	SE	*p* Value
PSQI total score	time (ref = pre)	−0.07	0.20	0.724
	group (ref = control)	0.23	0.26	0.360
	time × group	**−0.67**	**0.27**	**0.012**
Self-rated dormitory sleep environment	time (ref = pre)	−0.01	0.08	0.846
	group (ref = control)	−0.09	0.10	0.376
	time × group	0.13	0.11	0.215
Roommates’ influence on sleep schedule	time (ref = pre)	0.01	0.08	0.886
	group (ref = control)	0.22	0.10	0.033
	time × group	−0.21	0.11	0.066
Dormitory environment influence on sleep quality	time (ref = pre)	−0.08	0.08	0.310
	group (ref = control)	0.05	0.09	0.585
	time × group	−0.09	0.11	0.417

Note. SE = standard error; PSQI = Pittsburgh Sleep Quality Index.

**Table 4:** Linear mixed-effects models of intervention effect on secondary outcomes.

Secondary Outcomes		β	SE	*p* Value
PSQI—Subjective sleep quality	time (ref = pre)	0.13	0.06	0.040
	group (ref = control)	0.15	0.08	0.049
	time × group	−0.22	0.08	0.008
PSQI—Sleep latency	time (ref = pre)	−0.05	0.07	0.435
	group (ref = control)	0.02	0.09	0.787
	time × group	−0.02	0.09	0.799
PSQI—Sleep duration	time (ref = pre)	−0.07	0.06	0.180
	group (ref = control)	0.08	0.07	0.279
	time × group	−0.09	0.07	0.234
PSQI—Sleep efficiency	time (ref = pre)	0.04	0.04	0.379
	group (ref = control)	0.03	0.05	0.519
	time × group	−0.09	0.06	0.102
PSQI—Sleep disturbance	time (ref = pre)	0.07	0.05	0.111
	group (ref = control)	0.04	0.05	0.415
	time × group	−0.09	0.06	0.148
PSQI—Use of sleep medication	time (ref = pre)	0.10	0.03	0.001
	group (ref = control)	−0.03	0.03	0.302
	time × group	−0.06	0.04	0.172
PSQI—Daytime dysfunction	time (ref = pre)	−0.32	0.08	<0.001
	group (ref = control)	−0.07	0.08	0.441
	time × group	−0.07	0.10	0.507
Self-reflection of potential disturbance to roommate’s sleep	time (ref = pre)	−0.07	0.08	0.403
	group (ref = control)	0.21	0.10	0.037
	time × group	−0.15	0.11	0.162
Reaction to roommate’s reminder of disruptive behaviour	time (ref = pre)	0.22	0.08	0.006
	group (ref = control)	0.18	0.09	0.059
	time × group	−0.22	0.11	0.038
Dormitory conflict due to sleep disturbances	time (ref = pre)	−0.15	0.05	0.005
	group (ref = control)	−0.03	0.08	0.667
	time × group	0.11	0.07	0.125
Roommate relationship	time (ref = pre)	−0.19	0.05	0.001
	group (ref = control)	−0.28	0.09	0.002
	time × group	0.20	0.07	0.006
Sleep importance	time (ref = pre)	−0.11	0.06	0.071
	group (ref = control)	−0.02	0.07	0.718
	time × group	−0.02	0.08	0.798

Note. SE = standard error; PSQI = Pittsburgh Sleep Quality Index.

## Data Availability

The data presented in this study are available on request from the corresponding author after anonymisation. The data are not publicly available to ensure participant confidentiality.

## Supplementary Materials

The following supporting information can be downloaded at: https://www.mdpi.com/article/10.3390/ijerph192215337/s1, Table S1: Items of secondary outcome variables (except PSQI scores); Table S2: Items in the feedback survey for the intervention group; Table S3: Results of the sensitivity analysis.
