# Supplementary material for: Effectiveness of Multidomain Dormitory Environment and Roommate Intervention for Improving Sleep Quality of Medical College Students: A Cluster Randomised Controlled Trial in China
Source: Int J Environ Res Public Health. 2022 Nov 20;19(22):15337. doi: 10.3390/ijerph192215337 (PMC9691206; doi:10.3390/ijerph192215337)
Supplement: Supplementary file 1 [file ijerph-19-15337-s001.zip › ijerph-2003791-supplementary.pdf]

# Supplementary Materials

## Table S1. Items of secondary outcome variables (except PSQI scores).

| | | | | |
|---|---|---|---|---|
| 1. How do you get along with your roommates at present? | | | | |
| ① Very Bad | ② Bad | ③ Ordinary | ④ Good | ⑤ Very Good |
| 2. Have you considered the influence of your own behavior on your roommates when she or he was asleep in the last month? | | | | |
| ① Never | ② Occasionally | ③ Sometimes | ④ Often | ⑤ Always |
| 3. In the last month, if your behavior had affected your roommate's sleep, after you realised or were reminded by your roommate you would: | | | | |
| ① stop immediately | ② stop later | ③ continue to do, but be careful | ④ ignore him (her) | |
| 4. Has there been any conflict in your dormitory due to sleep problems in the last month? | | | | |
| ① Never | ② Occasionally | ③ Sometimes | ④ Often | ⑤ Always |
| 5. How important do you think sleep is? | | | | |
| ① Completely unimportant | ② Not very important | ③ Moderate | ④ Relatively important | ⑤ Very important |

## Table S2. Items in the feedback survey for the intervention group.

| | | | | |
|---|---|---|---|---|
| 1. After the intervention, what changes do you think in the sleeping environment of your dormitory? | | | | |
| ① Significantly got worse | ② A little worse | ③ No change | ④ Improved a little | ⑤ Significantly improved |
| 2. After the intervention, do you feel your sleep quality has changed? | | | | |
| ① Significantly got worse | ② A little worse | ③ No change | ④ Improved a little | ⑤ Significantly improved |
| 3. In general, can you accept this form of sleep intervention? | | | | |
| ① Completely unacceptable | ② Not very acceptable | ③ Reluctantly acceptable | ④ Acceptable | ⑤ Very acceptable |
| 4. Which intervention measures do you think are the most effective? (Multiple choices are allowed) | | | | |
| ① Health education (oral presentation+ brochures) | ② Making dormitory sleep rules | ③ Provide earplugs | ④ Provide eye mask | ⑤ Other measures, please explain:_____________ ________ |

**Table S3. Results of the sensitivity analysis.**

| Sensitivity analyses | Outcome variables | Interaction term: time*group | | |
|---|---|---|---|---|
| | | **β** | **SE** | **p value** |
| Linear mixed-effects models with no covariates | PSQI total score | -0.54 | 0.26 | 0.041 |
| | Self-rated dormitory sleep environment | 0.11 | 0.10 | 0.292 |
| | Roommates' influence on sleep schedule | -0.20 | 0.11 | 0.076 |
| | Dormitory environment influence on sleep quality | -0.06 | 0.11 | 0.544 |
| Adjusting for additional covariates | PSQI total score | -0.65 | 0.25 | 0.008 |
| | Self-rated dormitory sleep environment | 0.13 | 0.11 | 0.228 |
| | Roommates' influence on sleep schedule | -0.21 | 0.11 | 0.069 |
| | Dormitory environment influence on sleep quality | -0.08 | 0.11 | 0.480 |

*Note.* SE = standard error.
